# Primary urachal signet ring cell carcinoma: A case report

**DOI:** 10.3389/fonc.2022.1034245

**Published:** 2022-10-06

**Authors:** Qing Wang, Xiaolong Chen, Jianqing Zhang, Yuting Luo, Kehua Jiang

**Affiliations:** ^1^ Department of Urology, Guizhou Provincial People’s Hospital, Guiyang, China; ^2^ Zunyi Medical University, Zunyi, China

**Keywords:** case report, diagnosis, signet ring cell carcinoma, treatment, urachal carcinoma

## Abstract

Urachal signet ring cell carcinoma is a kind of rare but aggressive tumor, and a few cases have been reported previously. A 49-year-old male patient with primary complaints of increased frequency of urination, urodynia, and hematuria was diagnosed to have primary urachal signet ring cell carcinoma by our department. Multiple metastases were found in the sigmoid colon, terminal ileum, mesentery, and peritoneum during the operation, and palliative surgery involving partial cystectomy with en bloc resection of the urachus was then performed. A chemotherapy regimen of fluorouracil combined with cisplatin was made for this case. In addition, this patient also received anlotinib for targeted therapy. So far, this patient has done well on regular follow-up for 6 months and is in stable condition. We reported this additional urachal signet ring cell carcinoma case and conducted a literature review to strengthen our cognition of this disease.

## Introduction

Urachal carcinoma is a kind of rare but aggressive tumor, which is reported with an incidence of one case per million per year and accounts for <1% of bladder-associated malignancy (1). Adenocarcinoma is the most common urachal carcinoma (>90%), and about 20%–40% of bladder adenocarcinoma is of urachal origin (2). Signet ring cell carcinoma is a subtype of urachal adenocarcinoma and only accounts for 7% (3). A few cases regarding urachal signet ring cell carcinoma have been reported (4–13). In the current study, one additional case in a 49-year-old male patient was reported, and a literature review was conducted to strengthen the cognition of urachal carcinoma.

## Case presentation

**Figure 1:**
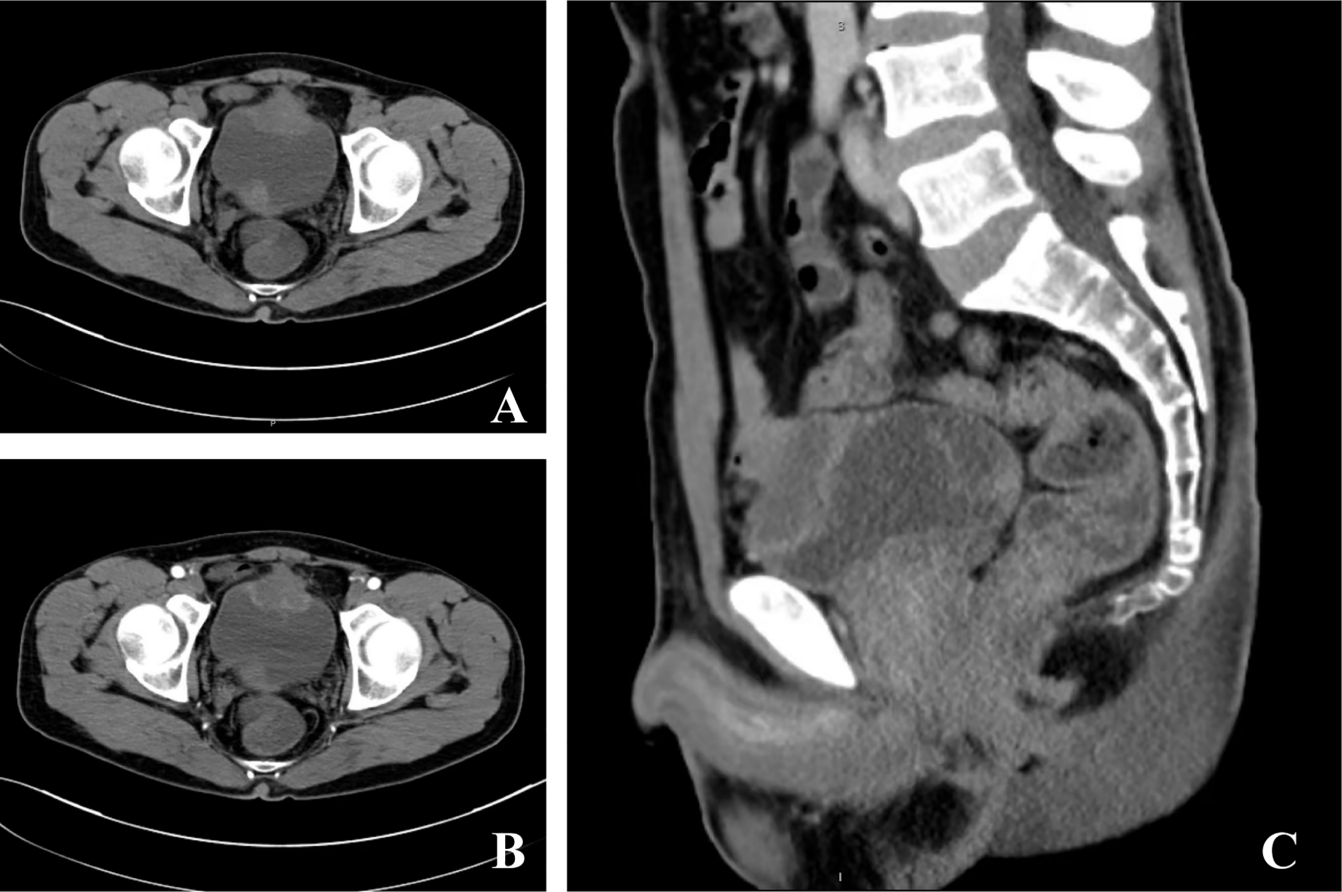
Computed tomography of the current urachal signet ring cell carcinoma case. **(A, B)** A tumor with the size of 4.0 cm × 2.7 cm × 4.0 cm and progressive annular enhancement was observed in the anterior wall of the bladder. The mass in the trigone of the bladder was confirmed to be a blood clot *via* cystoscopy. **(C)** The tumor extended superiorly up to just below the umbilicus.

A 49-year-old male patient was admitted to our department due to increased frequency of urination, urodynia, and hematuria for about 11 months. Ultrasonography identified a solid mass with the size of 3.7 cm × 2.6 cm in the anterior wall of the bladder. Computed tomography of the abdomen further showed that the bladder was irregularly thickened. A tumor with the size of 4.0 cm × 2.7 cm × 4.0 cm and progressive annular enhancement was observed in the anterior wall, which extended superiorly up to just below the umbilicus (**Figure 1**). Cystoscopy identified a cauliflower-like tumor with the size of 4 cm × 4 cm and a broad base on the top wall of the bladder. Diagnostic resection was then performed, and histological analysis revealed that there was bladder adenocarcinoma (predominantly consisting of signet ring cell type) in the excised mass. Immunohistochemistry showed that villin, CK20, CDX-2, and Ki-67 were positively stained in the tumor, while P40, P63, and CK7 were negatively stained. No gastrointestinal tumor was found by gastroscopy, colonoscopy, or gastrointestinal barium.

**Figure 2:**
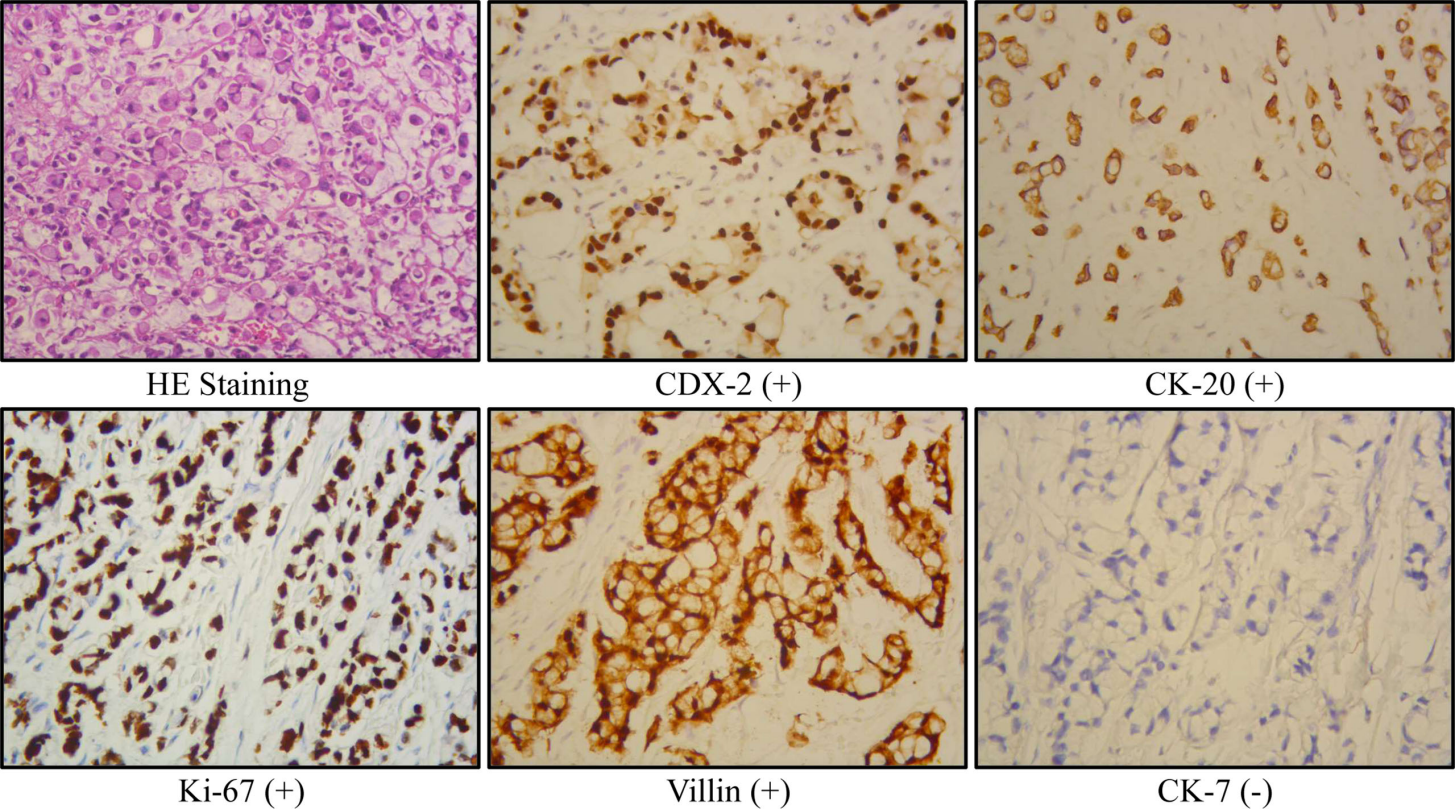
Representative immunohistochemical results of the current urachal signet ring cell carcinoma case.

Radical cystectomy was prepared for this patient according to the above findings. During the surgical exploration, a urachal mass extending from the umbilicus to the dome of the urinary bladder was found. Unfortunately, multiple metastases were seen in the sigmoid colon, terminal ileum, mesentery, and peritoneum, so palliative surgery including partial cystectomy and urachal mass cystectomy was conducted for this patient. Postoperative pathology showed that the tumor consisted of poorly to moderately differentiated adenocarcinoma, including signet ring cell carcinoma and mucinous adenocarcinoma (**Figure 2**). The carcinoma invaded the subserosal fibrous connective tissue and some area had broken through the serosa. The immunohistochemical results were as follows: CKpan (+), villin (+), CK20 (+), CDX-2 (+), β-catenin (+), SATB (+), cdh17 (+), CK7 (−), GATA-3 (−), CK5/6 (−), P40 (−), P63 (−), PAP (−), PSA (−), CK7 (−), and Ki-67 (+). In addition, the periodic acid–Schiff stain and mucin carmine staining were both positive in the carcinoma tissue. Postoperative PET-CT identified several metastases in the peritoneum and omentum. The clinical stage for this patient was finally referred to as IVb according to the Sheldon staging system.

**Figure 3:**
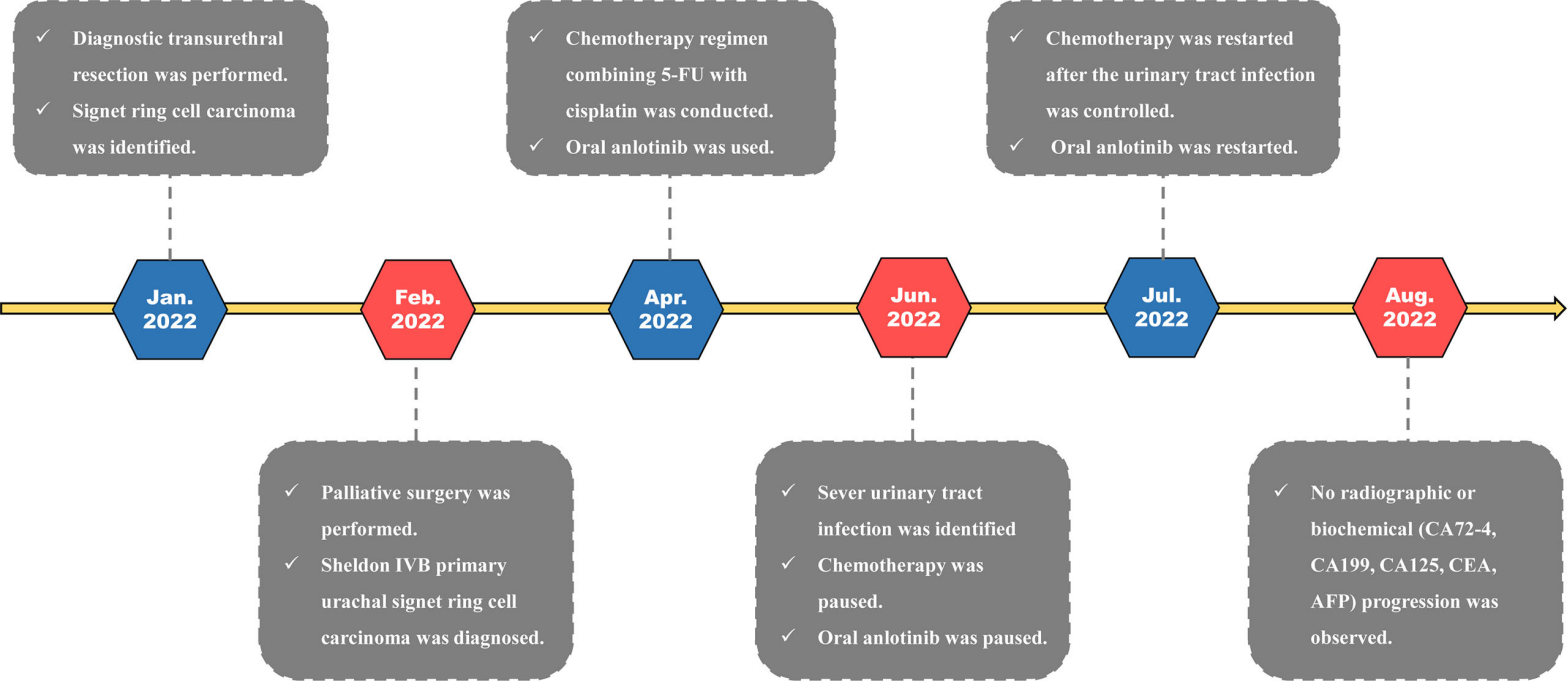
Treatment timeline of the current urachal signet ring cell carcinoma case.

Chemotherapy was conducted for this patient after surgery. A chemotherapy regimen (repeated every 21 days) involving fluorouracil (100 mg/m^2^, civ 96 h, days 1–4) combined with cisplatin (75 mg/m^2^, ivgtt, days 1–3) was made for this patient. In addition, the patient also received oral anlotinib (10 mg/day on days 1–14 of a 21-day cycle) for targeted therapy. So far, this patient has received 4 cycles of pharmaceutical therapy and is now doing well on regular follow-up. The treatment timeline for this patient is summarized in **Figure 3**.

Tumor biomarkers, including carcinoembryonic antigen (CEA), alpha-fetoprotein (AFP), carbohydrate antigen 72-4 (CA72-4), carbohydrate antigen 19-9 (CA19-9), and carbohydrate antigen 125 (CA125), were detected for this case. CEA was 1.2 ng/ml (normal: 0–5), AFP was 1.9 μg/L (normal: <7), CA72-4 was 11.3 U/ml (normal: 0–6.9), CA19-9 was 9.3 U/ml (normal: 0–39), and CA125 was 145 U/ml (normal: 0–35) in the first month after surgery. In the most recent follow-up, CEA was 1.3 ng/ml, AFP was 2.6 μg/L, CA72-4 was 12.8 U/mL, CA19-9 was 10.3 U/mL, and CA125 decreased to 22.2 U/ml.

## Discussion

The urachus is a lumen structure connecting the allantois and the bladder during embryonic development. After the third trimester, it obliterates to a 5–10-cm fibromuscular canal within the Retzius space and stretches from the bladder roof to the umbilicus (1). When the urachus obliterates incompletely, it may form a urachal fistula, urachal cyst, and urachal bladder diverticulum (14, 15). Actually, a microscopic residue of the urachus is found in 32% of adults even without the above malformations (16). The urachus can be histologically divided into three layers. The inner layer mainly consisted of the urothelium, while 30% is columnar epithelium or goblet cell (16). The middle layer is submucosal connective tissue and the outer is smooth muscle. Despite the common presence of urothelium in urachal residues, adenocarcinoma is the most common urachal carcinoma (>90%). Glandular metaplasia of the urachal urothelium is postulated to participate in the oncogenesis of urachal adenocarcinoma (17). Another theory suspects that urachal adenocarcinomas may arise from displaced cloacal epithelium (18).

Urachal carcinoma mainly affects patients aged 20–90 years and shows a male predilection (2). The most common symptom of urachal carcinoma is hematuria (2). Other symptoms, such as mucinuria, abdominal pain, urodynia, and dysuria, have also been reported (2, 19). The diagnostic criteria suggested by Gopalan et al. (17) are frequently used for primary urachal tumors, and the clinical classification can be assessed according to the Sheldon, Mayo, and TNM staging systems (20–22). It should be indicated that metastases from other sites (especially the gastrointestinal tract) must be ruled out before a primary urachal carcinoma can be diagnosed. In addition, it is also important to differentiate urachal adenocarcinoma from non-urachal adenocarcinoma of the bladder because they require different treatment strategies. Generally, urachal adenocarcinoma locates in the dome/anterior wall of the bladder and lacks widespread cystitis cystica/glandularis beyond the dome/anterior wall (20). In contrast, primary bladder adenocarcinoma often locates in the lateral/bottom wall of the bladder and presents with cystitis cystica/glandularis.

Partial cystectomy with en bloc resection of the urachus is now the primary surgical option for localized urachal cancer. Whether lymphadenectomy should be conducted remains controversial because lymph node positivity was found in only 17% of cases and lymphadenectomy contributes little to improvement in survival (2, 23). About 21% of urachal carcinoma patients have distant metastasis at first presentation (2). For patients with metastasis, systemic treatment is suggested. There is currently no standard chemotherapy regimen for urachal adenocarcinoma, but the chemotherapy regimens for bladder cancer and colorectal cancer can be referred. It is reported that the combination of 5-fluorouracil (5-FU) with cisplatin provides more benefit than 5-FU or cisplatin alone in metastatic urachal carcinoma (2). In addition, Goss et al. reported that one patient with urachal cancer had a transient 55% decrease in tumor size after receiving 800 mg/day of gefitinib, indicating that an epidermal growth factor receptor (EGFR) inhibitor seems to be an alternative choice for metastatic urachal carcinoma (24). In the current study, the combination of 5-FU with cisplatin and anlotinib is made for the patient and the condition keeps stable after surgery.

The reported median overall survival (OS) time from the diagnosis of urachal cancer in all stages ranges from 42.9 to 67 months, and the estimated 5-year OS rate is about 50% (2, 23). Sheldon stage >IIIB, Mayo stage >II, the presence of lymph node metastases or distant metastases, and tumor-positive surgical margins have been identified as independent parameters of poor prognosis (2). Although it is unknown whether there are differences in behavior among different urachal adenocarcinoma subtypes, urachal signet ring cell carcinoma often presents with high grade, high stage, and poor prognosis, which may be explained by its more diffuse infiltrative growth (3). We summarized the clinical information of previously reported urachal signet ring cell carcinoma cases in **Table 1**. As we can see, most patients are men (>80%) and all of them are diagnosed at a late stage (Sheldon ≥III). The OS time after surgery ranges from 6 to 72 months, and the 5-year OS rate is only about 22%. In the current study, no significant radiographic (assessed by CT and magnetic resonance imaging) or biochemical (CEA, AFP, CA72-4, CA19-9, and CA125) progression was observed in the patient during 6 months after surgery. Chemotherapy and regular follow-up will be continually conducted for this patient.

**Table 1:** Overview of previous case reports regarding primary urachal signet ring cell carcinoma.

Year	Authors	Nation	Case	Sex	Age (y)	Primary symptom	Stage	Treatments	Recurrence after surgery (m)	Survival after surgery (m)
1978	Jakse et al. (4)	Germany	1	Male	51	Dysuria	IIIA	Radical cystoprostatectomy with en bloc resection of the urachus	NA	>72
1981	Atsuo et al. (5)	Japan	1	Male	53	Hematuria	IIIA	Partial cystectomy with en bloc resection of the urachus, chemotherapy	20	35
1985	Gorrea et al. (6)	Spain	1	NA	28	Hematuria	IVB	Cytoreductive surgery	–	6
1990	Chen et al. (7)	USA	1	Male	73	Hematuria	IIIA	Partial cystectomy with en bloc resection of the urachus	NA	6
1997	Loggie et al. (8)	USA	1	Male	35	Hematuria	IVB	Cytoreductive surgery, chemotherapy	–	23
2004	Singh et al. (9)	India	2	Male	17	Hematuria	IIIA	Partial cystectomy with en bloc resection of the urachus, radiotherapy	21	NA
				Male	31	Hematuria	IVB	Cytoreductive surgery, chemotherapy	9	19
2007	Akihiro et al. (10)	Japan	1	Male	65	Hematuria	IIIA	Radical cystectomy with en bloc resection of the urachus, chemotherapy	6	>60
2009	Lars et al. (11)	Sweden	2	Male	51	Hematuria	IIIC	Radical cystoprostatectomy with en bloc resection of the urachus	9	14
				Male	53	Hematuria	IVA	Radical cystoprostatectomy with en bloc resection of the urachus, chemotherapy	16	26
2013	D. Hayes et al. (12)	Ireland	1	Female	38	Abdominal pain	IIIA	Partial cystectomy with en bloc resection of the urachus	NA	NA
2016	Amit et al. (13)	India	1	Male	61	Increased frequency of urination	IIIA	Radical cystectomy with en bloc resection of the urachus	NA	NA

Stage is accessed according to the Sheldon staging systems.

NA, not available; y, years; m, months.

## Conclusion

Urachal signet ring cell carcinoma is a kind of rare but aggressive tumor, which often presents with high grade and high stage. Despite the poorer prognosis, the diagnosis, classification, and treatment strategy of urachal signet ring cell carcinoma are currently in accordance with other urachal carcinomas. The early diagnosis method and standard high-level evidence guiding the treatment are lacking for urachal carcinoma. More significant efforts are needed to increase the cognition of this disease.

## Data availability statement

The raw data supporting the conclusions of this article will be made available by the authors, without undue reservation.

## Ethics statement

The studies involving human participants were reviewed and approved by Guizhou Provincial People’s Hospital ethics committee. The patients/participants provided their written informed consent to participate in this study. Written informed consent was obtained from the individual(s) for the publication of any potentially identifiable images or data included in this article.

## Author contributions

QW and XC drafted the manuscript. JZ and YL collected the clinical data. KJ did the surgery. All authors read and approved the final manuscript.

## Funding

This study was funded by the National Natural Science Foundation of China (Number: 82060462), Science and Technology Plan Project of Guizhou Province (Number [2019]:5405), and the Doctoral Foundation of Guizhou Provincial People’s Hospital (GZSYBS[2018]02).

## Conflict of interest

The authors declare that the research was conducted in the absence of any commercial or financial relationships that could be construed as a potential conflict of interest.

## Publisher’s note

All claims expressed in this article are solely those of the authors and do not necessarily represent those of their affiliated organizations, or those of the publisher, the editors and the reviewers. Any product that may be evaluated in this article, or claim that may be made by its manufacturer, is not guaranteed or endorsed by the publisher.
